# Study on the Relationship Between Confucian Filial Piety Culture and Chinese Youth’s Entrepreneurial Intention

**DOI:** 10.3389/fpsyg.2021.783399

**Published:** 2022-01-27

**Authors:** Zhao Lily, Kong Fanzhu, Yang Xiurang

**Affiliations:** ^1^Faculty of Electronic Information Engineering, Huaiyin Institute of Technology, Huaian, China; ^2^School of Management, Suqian University, Suqian, China

**Keywords:** Chinese Confucian filial piety culture, entrepreneurial intention, achievement motivation, self-efficacy, risk-taking tendency

## Abstract

As an important part of entrepreneurial environment, Culture directly or indirectly affects individual entrepreneurial intention. Taking young students as the survey object, this paper uses empirical research methods to explore the relationship between Confucian filial piety culture and Chinese youth’s entrepreneurial intention. The results show that there is a positive correlation between Confucian filial piety culture and Chinese youth’s entrepreneurial intention, achievement motivation, risk-taking tendency and self-efficacy; Confucian filial piety culture has not only a direct effect on Chinese youth’s entrepreneurial intention, but also an indirect effect with achievement motivation, self-efficacy and risk-taking tendency as intermediary variables.

## Introduction

Youth is the hope of the country and the nation. Entrepreneurship is an important way to promote economic and social development and improve people’s livelihood. The whole society should attach importance to and support young people’s innovation and entrepreneurship, provide more favorable conditions, and build a broader stage (Xi, 2013). In order to encourage Chinese youth to engage in innovation and entrepreneurship activities, the government has issued a number of supporting policies to create a good entrepreneurial environment, but the introduction of these supporting policies has not significantly improved the entrepreneurial participation rate and success rate of Chinese youth. It can be seen that there are still many factors restricting the development of Chinese youth entrepreneurship. Previous studies have shown that environment is one of the important factors affecting individual entrepreneurship, which restricts or promotes individual entrepreneurship (Chen and Zhao, 2019). Economic environment, institutional environment and cultural environment are the most important dimensions. Existing researches mainly related to the impact of the economic and institutional environment on individual entrepreneurial intention, and these studies have achieved significant results (Kong and Zhao, 2018; Klein et al., 2019). Among them, some scholars have studied the relationship between cultural environment and entrepreneurship, but most of them take entrepreneurial culture or entrepreneurial atmosphere as the main variables, while there are few studies on entrepreneurial culture from the perspective of Chinese traditional culture (Burt and Burzynska, 2017; Sha and Ding, 2021).

The Chinese nation has a continuous civilization history of more than 5,000 years and has created a broad and profound Chinese culture (Xi, 2018). Chinese traditional culture has accumulated the deepest spiritual pursuit of the Chinese nation, and contains rich spiritual concepts and philosophical wisdom such as patriotism, striving and enterprising, morality and self-cultivation, respecting teachers and education. The Chinese excellent traditional culture represented by Confucianism has a proven historical calculation in the culture of behavior habits (Jin, 2020). Confucian filial piety, as the gene of Chinese traditional culture, has been implicitly implanted into China’s social and political structure and Chinese people’s lifestyle and spiritual temperament (Liu, 2019). Xi (2018) also stressed that the fine traditional culture of the nation has always exerting a subtle influence on the way of thinking and behavior of the Chinese people At some point, Chinese children grow up under filial piety education, and their behavior patterns will inevitably be affected by filial piety culture. However, most Chinese researchers do not pay enough attention to Chinese traditional culture, overemphasize the impact of economic and institutional environment on entrepreneurship, and ignore the power of Chinese traditional culture. Because of this, this paper takes the Confucian filial piety culture as the background, selects college students who are convenient to contact and investigate as the research object, analyzes the impact of the Confucian filial piety culture on young people’s entrepreneurial intention., so as to explore the role of Chinese traditional culture on the entrepreneurial will of Chinese youth.

Entrepreneurship is a systematic project. Although it is an individual decision, the decision-making process is affected by many factors. This paper chooses Chinese Confucian filial piety culture to explore its role in individual entrepreneurial intention. The contribution of this paper mainly lies in the following two aspects. First, it preliminarily discusses the mechanism of Confucian filial piety culture on the individual entrepreneurial intention of Chinese youth. Culture will not only directly affect the individual behavior decision-making mode, but also affect individual decision-making through family environment. The second is to study the practical problems of Chinese Entrepreneurship from the perspective of traditional culture. Almost the behavior patterns of Chinese all over the world are inevitably affected by Chinese traditional culture. The role of this cultural element can’t be ignored in the study of current problems. This paper enriches the research results in this field.

## Literature Review

### Confucian Filial Piety Culture

Traditional Chinese society is based on filial piety, so filial piety is widely respected by people. Filial piety is not only the cornerstone of Chinese ethics, but also the spiritual basis for the formation of Chinese politics and law. After thousands of years, the ethics with filial piety as the core has been deeply rooted in the Chinese nation. It still has important value in today’s society. It plays an important role in the construction of contemporary ideology and culture, and has an important impact on people’s behavior, the generation of emotion and the formation of thinking. Filial piety is one of the important contents of Chinese traditional culture. Chinese traditional social structure takes the family as a basic unit. An important value of filial piety is to maintain the harmony, unity and happiness of the family. Zhao (2014) summarized that the content of Confucian filial piety culture includes eight aspects: raising relatives, respecting relatives, obeying relatives, admonishing relatives, returning relatives, extending relatives, following relatives, and showing relatives. Confucian filial piety emphasizes supporting parents based on blood relationship, that is, children should support their parents. Wang (2002) believes that both filial piety and righteousness are ideas vigorously advocated in the Confucian filial piety culture, emphasizing that people should be loyal to their country and respect their relatives. With the changes of the times, this concept has gradually evolved into a personal achievement thought for fame and wealth or glory. Gan and Feng (2020) found that the Confucian filial piety makes children show the concept of “showing their parents.” Children get more love from their parents and bear higher expectations, so they show a stronger willingness to repay their parents. Taken together, children’s support and respect for their parents are the basis of filial piety. Child generation should not only provide their parents with basic material needs, but also meet their spiritual needs. Through their continuous efforts, they can make achievements in a certain field and make their parents feel proud.

### Entrepreneurial Intention and Influencing Factors

Intention is a psychological vocabulary, which usually refers to an individual’s views or thoughts on things. Intention can be divided into will and wish. Will refers to the mind and the direction of the heart; Wish refers to desire and motive force. Therefore, intention is to achieve a specific goal and direction, and then use your ability to achieve that goal and direction. Entrepreneurial intention is the product of the integration of entrepreneurship and psychology. With the development of entrepreneurship, entrepreneurial intention is gradually used to measure the entrepreneurial aspirations of entrepreneurial individuals or organizations. In the field of individual entrepreneurship research, entrepreneurial intention usually refers to a subjective attitude of potential entrepreneurs to engage in entrepreneurial activities (Kong et al., 2019).

The formation of entrepreneurial intention is affected by many factors, including not only the internal factors such as entrepreneurs’ own characteristics and previous entrepreneurial experience, but also external factors such as economy, society and policy. Wilken (1979) found that the need of self-actualized and the self-confidence and desire of individuals have a significant impact on the entrepreneurial intention by analyzing the personality differences between entrepreneurs and non-entrepreneurs. The higher the need of self-actualized and self-confidence of individuals, the easier it is to produce positive entrepreneurial intention. Individual entrepreneurial ability, financial support, support provided by the school, encouraging policies issued by the government and macroeconomic environment have a certain impact on College Students’ entrepreneurial intention. Shen et al. (2013) found that college students can learn entrepreneurial knowledge, improve their entrepreneurial ability and generate entrepreneurial intention by receiving entrepreneurship education in schools. Dou and Bao (2016) believes that the entrepreneurial knowledge and experience accumulated by individuals from their previous entrepreneurial experience will affect their subsequent entrepreneurial intention and behavior.

Based on the above analysis, we can find that entrepreneurial intention was affected by many different factors. As a combination of behavior mode and psychological thinking, personality characteristics can directly affect the generation of entrepreneurial intention. Meanwhile, the impact of cultural environment on people will be reflected in the formation of individual personality characteristics. Therefore, as a bridge connecting traditional culture and entrepreneurial intention, the research on personality characteristics is of positive significance. According to the collation and induction of literature, this paper summarizes personality characteristics into three dimensions: achievement motivation, self-efficacy and risk-taking tendency.

## The Relationship Between Confucian Filial Piety Culture and Chinese Youth’s Entrepreneurial Intention

### Confucian Filial Piety Culture and Entrepreneurial Intention of Chinese Youth

Confucian filial piety culture includes supporting, respecting and inheriting parent’s ambition. Supporting is the lowest level of filial piety, the basic requirement of filial piety, and ensures the material needs of parents. However, maintenance is only reflected in the material level, which can’t reflect the humanistic characteristics of filial piety. Filial piety is not only to be able to support, but also to respect parents. Inheriting ambition is the highest level of filial piety and the inheritance of reasonable politics. Existing studies also show that children whose parents are entrepreneurs are more likely to start businesses in the future. China is experiencing economic transition, and innovation and entrepreneurship are more important than ever before. Therefore, the state has successively issued many policies and measures to promote Innovation & Enterprise and protect entrepreneurship, and the social attitude toward entrepreneurship have gradually changed. Nowadays, independent entrepreneurship has not only become an important way for college students to realize their self-worth, but also a successful entrepreneurship has become a way to honor their elders. Successful entrepreneurs such as Jack Ma, Tao Huabi, Pony Ma, and Ren Zhengfei have been recognized and respected by the state, society and villagers, bringing glory to their families. Therefore, we may believe that Chinese youths with filial piety are more willing to try entrepreneurship in order to make their parents live a better life and add luster to their parents. Therefore, this paper puts forward the following hypothesis:

H1: Confucian filial piety culture is positively correlated with Chinese youths’ entrepreneurial intention.

### Confucian Filial Piety Culture and Personality Characteristics of Chinese Youth

As an internal driving force, achievement motivation will encourage individuals to make continuous efforts to achieve success. Confucian filial piety plays an important guiding role in people’s moral cultivation and the formation of code of conduct. After thousands of years, filial piety is still an important code of conduct and an important standard to measure people. The thought of “settling down and walking the Tao and becoming famous in the world is the destination of filial piety” was put forward in the book of filial piety. That is, individuals should continue to work hard, make achievements in their fields, become famous and shine in the family. Under the influence of this concept, Chinese youths have a strong desire to succeed and show their parents. Therefore, we propose the following hypothesis:

H2a: Confucian filial piety culture is positively correlated with Chinese youth achievement motivation.

Self-efficacy is an individual’s confidence and expectation that he can effectively complete a task. It is an individual’s self-evaluation of his own ability (Yang et al., 2021). Confucian filial piety not only advocates respecting and supporting parents, but also inheriting parents, that is, children inherit their parents’ correct and reasonable wishes and career. The formation of children’s values is deeply influenced by family education, and family education influenced by filial piety culture is full of the thought of merit and fame. It emphasizes that the purpose of learning is to shine on the family. As a result, most parents define the quality of a job by social status, fame, wealth and reputation, which will virtually enhance their children’s confidence and expectation in completing a job. Therefore, we propose the following hypothesis:

H2b: Confucian filial piety culture is positively correlated with Chinese youth self-efficacy.

Risk-taking tendency refers to an individual’s acceptance of risk when making decisions. Confucian filial piety has always advocated supporting parents based on blood relationship, and the most basic thing to support parents is to give them corresponding help at the economic and material level. The concept of being kind to parents formed by Chinese youth from childhood has been deeply rooted in their minds. The idea of supporting parents with better material security and living environment through their own efforts after growing up has a positive effect on the formation of individual risk-taking tendency, which will make them not afraid of risks and dare to challenge all kinds of difficulties. Based on this, we propose the following hypothesis:

H2c: Confucian filial piety culture is positively correlated with Chinese youth’s risk-taking tendency.

### Personality Characteristics and Entrepreneurial Intention

Achievement motivation is the internal drive of individuals to actively and unremittingly complete an activity in order to achieve a certain goal. People with stronger achievement motivation are often more actively face various difficulties and challenges. They are also more impossible make some changes and innovations, and try their best to achieve their goals, so as to obtain higher achievements and glory. Liu (2017) found that College Students’ personality characteristics is positively correlated with entrepreneurial effectiveness, and the internal motivation of college students can induce them to make some behavior. Wu (2018) found that improving students’ achievement motivation can stimulate their desire for success, make them sprout entrepreneurial ideas and be more willing to devote themselves to entrepreneurship. Based on the above analysis, we propose the following hypothesis:

H3a: Achievement motivation is positively correlated with entrepreneurial intention.

Self-efficacy refers to the confidence that you can achieve your goals and successfully complete tasks. Many studies have shown that people with high self-efficacy can produce higher job performance. Ordinarily, the youth have higher self-efficacy than other groups, they can better complete challenging tasks and have a stronger willingness to start a business. Ding et al. (2009) found that entrepreneurial self-efficacy has a positive and direct relationship with entrepreneurial intention. Ye and Zhu (2019) proposed that individuals with personality characteristics such as self-confidence, independent spirit and sense of cooperation, firm will and sharp judgment actively engage in entrepreneurship. Accordingly, we propose the following hypothesis:

H3b: Self-efficacy is positively correlated with entrepreneurial intention.

Risk-taking tendency refers to the individual’s acceptance of risk and the tendency to take risks. Individuals with high risk-taking tendency have more courage to take risks. When they face risks, they often choose to accept rather than avoid. Entrepreneurship is an activity with high risk and high return. If an individual is willing to bear the high risk, he will have the opportunity to obtain the high return brought by entrepreneurship, and he will engage in entrepreneurial activities. Glaeser et al. (2009) believes that entrepreneurs are people who dare to take risks. Based on this, we can make the following hypothesis:

H3c: Risk-taking tendency is positively correlated with entrepreneurial intention.

### The Intermediary Role of Personality Characteristics Between Confucian Filial Piety Culture and Chinese Youth’s Entrepreneurial Intention

Confucian filial piety plays a great role in promoting family harmony and supporting spiritual belief. Filial piety belief can guide individuals to make certain actions according to the connotation and principles of filial piety. Influenced by the Confucian filial piety, young people are eager to provide better material conditions for their parents. This will lead them to have high entrepreneurial willingness. Although Confucian filial piety has always advocated the idea of parents’ standard, it does not advocate foolish filial piety, but pay attention to “strategic filial piety.” Children can advise their parents’ orders and discuss with their parents on some important matters. In this case, children’s decision-making is often encouraged and supported by their parents, which leads them to be full of self-confidence and positive action, which makes them have a high sense of self-efficacy and enthusiasm for entrepreneurship. Furthermore, Confucian filial piety emphasizes standing up and becoming famous, so that they are not afraid of failure, have a high tendency to take risks, and are more willing to carry out entrepreneurial activities. It can be seen that personality characteristics may play a mediating role between Confucian filial piety and entrepreneurial intention. Based on this, this paper puts forward the following hypothesis:

H4a: achievement motivation plays an intermediary role between Confucian filial piety culture and Chinese youth’s entrepreneurial intention;
H4b: self-efficacy plays an intermediary role between Confucian filial piety culture and Chinese youth’s entrepreneurial intention;
H4c: risk-taking tendency plays an intermediary role between Confucian filial piety culture and Chinese youth’s entrepreneurial intention.

**FIGURE 1:**
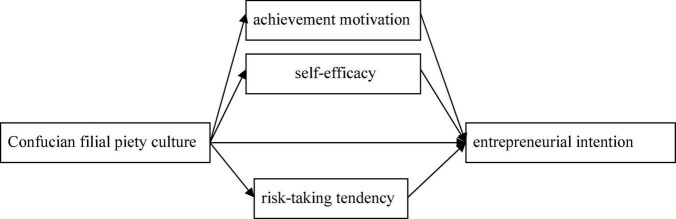
Research framework.

The above hypothesis constitute the research framework of this paper (see Figure 1).

## Variable Measurement and Data Collection

### Variable Measurement

In order to measure the Confucian filial piety culture, this paper sets up three items based on the study of Wu (2012) and Jiang (2013). On the base of research conclusion of Zhan (2008), this paper uses four items to measure individual achievement motivation. This paper designs six items to measure individual self-efficacy based on the study of Tang (2009). This paper adopts the research conclusions of Qian (2007), and uses four items to measure individual risk-taking tendency. For the measurement of entrepreneurial intention, three items are set based on the research results of Qian (2007) and Jiang (2013) (see Table 1).

**TABLE 1:** variable and measurement items.

Variable	Measurement items
Confucian filial piety culture	1. In order to make my parents live a better life, I will choose entrepreneurship. 2. For the family happiness, I am willing to devote time and energy to entrepreneurship. 3. My parents have certain requirements for my career development.
Achievement motivation	1. I don’t think finding a stable job is more important than becoming an entrepreneur. 2. I will persevere in solving the problems I am not sure to solve. 3. I will become more confident when I finish a very difficult job. 4. In case of failure, I will not lose the confidence and courage to achieve my goals.
Self-efficacy	1. I am good at analyzing the external environment to find opportunities and potential problems. 2. I like to solve problems in my own way. 3. I am willing to accept new things. 4. I can put forward new ideas and suggestions. 5. I have strong innovation ability. 6. I’m used to thinking twice before I make a decision.
Risk-taking tendency	1. I am not a routine. 2. For higher career achievements, I dare to take some risks. 3. I like challenging work. 4. For my future career, I tend to those with high risks and benefits.
Entrepreneurial intention	1. I had the idea of starting a business. 2. I have learned about entrepreneurship. 3. I am very eager to create my own enterprise.

### Data Collection

On the basis of the variable measurement items, this paper designs a questionnaire to collect the information of Confucian filial piety culture and Chinese youth’s entrepreneurial intention. When designing the questionnaire, we add two variables: gender and grade to measure personal information. A total of 300 questionnaires were distributed in this survey. When distributing the questionnaires, the respondents should be balanced in terms of gender and grade to the greatest extent, so as to ensure the effectiveness of the research results 206 valid questionnaires were finally recovered, and the recovery rate was 68.7%.

## Reliability and Validity Test

### Reliability Test

This paper uses spss25.0 and Cronbach α the reliability coefficient index tests the reliability of the questionnaire, and the results are shown in Table 2.

**TABLE 2:** Questionnaire reliability test results.

Variable	Confucian filial piety culture	Achievement motivation	Self-efficacy	Risk-taking tendency	Entrepreneurial intention
α	0.856	0.896	0.901	0.887	0.823

It can be seen from Table 2 that the reliability coefficient of each variable is greater than 0.8, which meets the statistical requirements, indicating that the scale has good reliability.

### Validity Test

The validity test results of entrepreneurial intention is shown in Table 3. The commonality of all items are higher than 0.4, indicating that the item can be extracted effectively. The value of KMO is 0.736, greater than 0.6, which means that the data is valid. Bartlett’s spherical test value is 392.4693 and the degree of freedom is 3, reaching the significance level, indicating that the sample is suitable for factor analysis. The cumulative variance of the factor is 79.004%, and the cumulative variance after rotation is 76.025 > 50%, which means that the items can be extracted effectively. Based on this, it can be considered that the validity analysis of entrepreneurial intention passed.

**TABLE 3:** Validity test results of entrepreneurial intention.

	Factor load	Commonality
	Factor1	
I had the idea of starting a business	0.878	0.768
I have learned about entrepreneurship	0.886	0.801
I am very eager to create my own enterprise	0.825	0.769
Characteristic root	2.327	–
Variance%	77.256%	–
Cumulative variance%	77.256%	–
Characteristic root (rotation)	2.286	–
Variance % (rotation)	77.126%	–
Cumulative variance % (rotation)	77.126%	–
KMO	0.741	–
Bartlett spherical test	352.652	–
Degree of freedom	3	–

The validity of other variables is also tested by the same method, and the test results meet the statistical requirements, which will not be repeated here.

## Empirical Test Results

### Correlation Analysis

In this paper, the person coefficient is used to test the correlation between variables. The result is expressed in R. The higher the R, the higher the correlation between the variables. When R is negative, it indicates that there is a negative correlation between variables; When R is positive, it indicates that there is a positive correlation between variables. The results are shown in Table 4.

**TABLE 4:** Correlation between the variables.

	Confucian filial piety	Achievement motivation	Self-efficacy	Risk-taking tendency	Entrepreneurial intention
Confucian filial piety	1				
Achievement motivation	0.573**	1			
Self-efficacy	0.568**	0.576**	1		
Risk-taking tendency	0.421**	0.567**	0.536**	1	
Entrepreneurial intention	0.556**	0.535**	0.513**	0.458**	1

**0.01.

As can be seen from Table 4, there is a significant correlation between all variables, and regression analysis can be carried out. In order to test the relationship between variables, we constructed regression models corresponding to hypothesis H1, H2a, H2b, H2c, h3a, H3b, and H3c, named model 1, model 2a, model 2b, model 2c, model 3a, model 3b, and model 3c. Simple linear regression method is adopted, and the regression results are shown in Table 5.

**TABLE 5:** Result of simple linear regression.

	Model 1	Model 2a	Model 2b	Model 2c	Model 3a	Model 3b	Model 3c
β	0.463**	0.435**	0.402**	0.328**	0.457**	0.425**	0.317**
*R* ^2^	0.778	0.781	0.775	0.689	0.717	0.771	0.711
F	671.23**	677.55**	655.68**	612.26**	610.23**	622.75**	702.33**

**p < 0.01.

It can be seen from Table 5 that the values of *F* test of all models are less than 0.05, indicating that the regression model is significant. Therefore, hypothesis H1, H2a, H2b, H2c, H3a, H3b, and H3c are verified.

### Mediation Role of Personality Characteristics

This paper uses the method proposed by Baron and Kenny (1986) to test the mediating effect of personality characteristics between Confucian filial piety culture and entrepreneurial intention.

### Mediation Role of Achievement Motivation

In order to test the intermediary role, this paper makes a regression analysis between Confucian filial piety culture, achievement motivation and entrepreneurial intention. The specific steps are as follows: firstly, regression analysis is carried out on the independent variable Confucian filial piety culture and the intermediary variable achievement motivation; Then, regression analysis is made on the intermediate variable achievement motivation and dependent variable entrepreneurial intention; Finally, the intermediary variable achievement motivation is added, and then the independent variable Confucian filial piety culture and the dependent variable entrepreneurial intention are regressed. The analysis results are shown in Table 6. After adding the intermediary variable of achievement motivation, the influence of Confucian filial piety culture on entrepreneurial intention is still significant, but the significance is significantly reduced, indicating that achievement motivation plays a partial intermediary role in the relationship between Confucian filial piety and Chinese youth entrepreneurial intention. Therefore, hypothesis H4a is verified.

**TABLE 6:** Mediation role of achievement motivation.

Variable	Regression 1	Regression 2	Regression 3	
	Achievement motivation	Entrepreneurial intention	Entrepreneurial intention	
			Step 1	Step 2
Confucian filial piety	0.435**		0.463**	0.255*
Achievement motivation		0.457**		0.132*
*R* ^2^	0.781	0.717	0.778	0.789
F	677.55**	610.23**	671.23**	486.57**

*p < 0.05 and **p < 0.01.

### Mediation Role of Self-Efficacy

Using the same method, the mediating effect of self-efficacy is tested, and the results are shown in Table 7. After adding the mediating variable of self-efficacy, the effect coefficient of Confucian filial piety on entrepreneurial intention is reduced from 0.463 to 0.262. The impact is still significant, but the effect path coefficient and significance are significantly reduced, indicating that self-efficacy plays a partial mediating role in the relationship between Confucian filial piety and Chinese youths’ entrepreneurial intention. Therefore, hypothesis H4b is verified.

**TABLE 7:** Mediation role of self-efficacy.

Variable	Regression 1	Regression 2	Regression 3	
	Self-efficacy	Entrepreneurial intention	Entrepreneurial intention	
			Step 1	Step 2
Confucian filial piety	0.402**		0.463**	0.262*
Self-efficacy		0.425**		0.173*
*R* ^2^	0.775	0.771	0.778	0.728
F	655.68**	622.75**	671.23**	478.76**

*p < 0.05 and **p < 0.01.

### Mediation Role of Risk-Taking Tendency

Similarly, the test results of the mediating effect of risk-taking tendency are shown in Table 8. After adding the intermediary variable of risk-taking tendency, the effect coefficient of Confucian filial piety on entrepreneurial intention is reduced from 0.463 to 0.197, and the impact is still significant, but the effect path coefficient and significance are significantly reduced, indicating that risk-taking tendency plays a partial intermediary role in the relationship between traditional filial piety and Chinese youths’ entrepreneurial intention. Therefore, hypothesis H4c is verified.

**TABLE 8:** Mediation role of risk-taking tendency.

Variable	Regression 1	Regression 2	Regression 3	
	Risk-taking tendency	Entrepreneurial intention	Entrepreneurial intention	
			Step 1	Step 2
Confucian filial piety	0.328**		0.463**	0.197*
Risk-taking tendency		0.317**		0.102*
*R* ^2^	0.689	0.711	0.778	0.779
F	612.26**	702.33**	671.23**	458.73**

*p < 0.05 and **p < 0.01.

## Research Conclusion and Discussion

Entrepreneurship is not only an individual activity, but also a social activity, which will be affected by many external factors such as social environment. To some extent, national culture determines personal values, thinking ideas and behavior. It is an important part of the entrepreneurial environment that can’t be ignored. China is an ancient civilization with a history of more than 5,000 years. In the long historical evolution, it has formed an excellent traditional culture that continues to today, affecting the thoughts and behaviors of all Chinese. It is more practical value to study China’s practical problems based on China’s cultural environment. Therefore, this paper selects the Confucian filial piety culture in Chinese traditional culture and uses empirical research methods to explore its impact on Chinese youths’ entrepreneurial intention. The results show that there is a significant positive correlation between Confucian filial piety and Chinese youths’ personality characteristics. Confucian filial piety culture has a positive impact on young people’s individual characteristics. The higher the level of filial piety, the higher their achievement motivation, self-efficacy and risk-taking tendency. From the regression coefficient, compared with self-efficacy and risk-taking tendency, the influence of Confucian filial piety culture on youth achievement motivation may be more obvious. This may be related to the expectations of parents for their children from childhood. Looking forward to their children’s success is almost every Chinese parent’s expectation and wish for their children, and children influenced by the culture of filial piety will take living up to their parents’ expectations as their standard of filial piety. Therefore, children who grow up in such a family environment have high achievement motivation. The study also shows that there is a significant positive correlation between Chinese youth’s personality characteristics and entrepreneurial intention. Individuals with high achievement motivation have higher expectations for their achievements and are more willing to go all out to achieve their ideals and goals. Therefore, they are easier to become entrepreneurs and hope to obtain material and spiritual satisfaction through entrepreneurship. Individuals with high self-efficacy are more confident in themselves, and can produce higher work performance in completing many types of tasks, so their entrepreneurial intention is higher. Individuals with risk-taking tendency like challenging tasks, are not afraid of risks, have more active thinking and more innovative ability, so they will have a stronger willingness to start a business. From the correlation and regression coefficient, the impact of achievement motivation on young people’s entrepreneurial intention is the most obvious, followed by self-efficacy and risk-taking tendency. This conclusion is basically consistent with the existing research results.

Through the above multi-level regression analysis, it can be seen that Confucian filial piety culture has not only a direct effect on Chinese youth’s entrepreneurial intention, but also an indirect effect with personality characteristics such as achievement motivation, self-efficacy and risk-taking tendency as intermediary variables. If young people have a thorough understanding of Confucian filial piety culture and agree to practice it, they will have a higher willingness to start a business. Moreover, Confucian filial piety culture will also imperceptibly affect young people’s achievement motivation, self-efficacy and risk-taking tendency, and then affect their entrepreneurial intention. Therefore, we can appropriately increase Confucian filial piety and other traditional cultural education, improve the cultural cultivation level of youth groups, enhance cultural self-confidence, build strong cultural influence, and then promote them to form positive personality characteristics. This will not only have a positive effect on entrepreneurial willingness, but also have a positive effect on young people engaged in other work.

Entrepreneurship is a long-term process full of thorns. In this process, the behavior of entrepreneurs may change with the development of entrepreneurial environment and entrepreneurial practice. For entrepreneurial individuals, the entrepreneurship may also be a process of self-growth. With the change of their own experience, their understanding of entrepreneurship and the intensity of entrepreneurial intention will change. However, this study uses cross-sectional data and does not consider this change, which may make the research results inconsistent with the reality of entrepreneurship, In the future research, we can use the dynamic research method to track the entrepreneurs. Due to the limited ability of personal research, this paper used the stepwise regression for testing the mediating analysis. This method has some limitations which may affect the accuracy of the research results. In the future, preacher and hayes technique can be used for testing the mediating analysis. In addition, due to the influence of COVID-19, the scope of data investigation in this paper is relatively small, and the diversity of samples is insufficient, which may make the research results not representative. At the same time, the manifestations of filial piety culture in different regions of China often show significant differences. This study does not consider this difference. In the future, we can make a comparative study of regional differences in this field.

## Data Availability Statement

The original contributions presented in the study are included in the article/supplementary material, further inquiries can be directed to the corresponding author.

## Author Contributions

KF and ZL: determined the overall research idea and structure of the manuscript. KF: literature review, theoretical assumptions and definition of key concepts. ZL and YX: questionnaire design, the distribution and recovery of questionnaires, the classification and sorting of survey data. All authors contributed to the article and approved the submitted version.

## Conflict of Interest

The authors declare that the research was conducted in the absence of any commercial or financial relationships that could be construed as a potential conflict of interest.

## Publisher’s Note

All claims expressed in this article are solely those of the authors and do not necessarily represent those of their affiliated organizations, or those of the publisher, the editors and the reviewers. Any product that may be evaluated in this article, or claim that may be made by its manufacturer, is not guaranteed or endorsed by the publisher.
